# Beneficial Effects of *Potentilla discolor* Bunge Water Extract on Inflammatory Cytokines Release and Gut Microbiota in High-Fat Diet and Streptozotocin-Induced Type 2 Diabetic Mice

**DOI:** 10.3390/nu11030670

**Published:** 2019-03-20

**Authors:** Lihua Han, Tiange Li, Min Du, Rui Chang, Biyuan Zhan, Xueying Mao

**Affiliations:** 1 Beijing Advanced Innovation Center for Food Nutrition and Human Health, College of Food Science & Nutritional Engineering, China Agricultural University, Beijing 100083, China; HannaH_19930216@163.com (L.H.); 13126750913@163.com (T.L.); rita219@126.com (R.C.); zhanby0122@163.com (B.Z.); 2 Key Laboratory of Functional Dairy, College of Food Science and Nutritional Engineering, Ministry of Education, China Agricultural University, Beijing 100083, China; 3 Department of Animal Sciences, Washington State University, Pullman, WA 99164, USA; min.du@wsu.edu

**Keywords:** *Potentilla discolor* Bunge water extract, diabetes, inflammation, gut microbiota, intestinal mucosal barrier function, short chain fatty acids

## Abstract

*Potentilla discolor* Bunge (PDB), a perennial herb, has been used as a traditional Chinese medicine in the therapy of many diseases. The aim of the current study was to investigate the effect of PDB water extract on systemic inflammation and gut microbiota in type 2 diabetic (T2D) mice induced by high-fat diet (HFD) and streptozotocin (STZ) injection. C57BL/6J mice were randomly divided into a normal diet (ND) group, T2D group, and PDB group (diabetic mice treated with PDB water extract at a dose of 400 mg/kg body weight). Results showed that PDB significantly decreased the levels of lipopolysaccharide (LPS) and pro-inflammatory cytokines in serum. Further investigation showed that PDB significantly reduced the ratio of *Firmicutes*/*Bacteroidetes* and the relative abundance of *Proteobacteria* in fecal samples of diabetic mice. In addition, PDB notably alleviated intestinal inflammation as evidenced by decreased expression of toll-like receptor 4 (TLR4), myeloid differentiation factor 88 (MyD88), nuclear factor-κB (NF-κB), and inflammatory cytokines. PDB also reversed the decreased expression of intestinal mucosal tight junction proteins including Claudin3, ZO-1, and Occludin. Meanwhile, the levels of fecal acetic acid and butyric acid and their specific receptors including G-protein-coupled receptor (GPR) 41 and 43 expression in the colon were also increased after PDB treatment. Our results indicated that PDB might serve as a potential functional ingredient against diabetes and related inflammation.

## 1. Introduction

Type 2 diabetes (T2D), a chronic systemic progressive metabolic disease, is characterized by hyperglycemia and chronic low-grade inflammation. Gut microbiota is a complex community of bacteria residing in the gastrointestinal tract, which usually maintains a mutually beneficial relationship with its host [1]. Numerous literatures revealed that individuals with T2D suffered from microbiota dysbiosis with alterations both in diversity and composition [2,3], indicating a potential relationship between gut microbiota and T2D. 

Lipopolysaccharide (LPS), a component present in the outer membrane of gram-negative bacteria, is considered as a trigger in an inflammatory response with the release of pro-inflammatory cytokines in blood and tissues, eventually resulting in insulin resistance and diabetes [4]. It has been proven that diabetic individuals possess a high quantity of gram-negative bacteria, especially those belonging to the phylum *Proteobacteria* [3]. The gut barrier is an indispensable structure to avoid the excessive translocation of LPS into the circulation system, thereby maintaining a mutually beneficial relationship between gut bacteria and host. It has been speculated that dysbiosis of gut microbiota might account for greater intestinal epithelium permeability in T2D patients, which consequently increased the leakage of LPS into the circulatory system [5,6]. Oral ingestion of *Akkermansia muciniphila*, an intestinal mucin-degrading bacterium, not only enhanced glucose tolerance but also alleviated white adipose tissue inflammation in high-fat diet (HFD) fed mice [7]. All this evidence suggests that gut microbiota might be a promising and feasible target for T2D treatment. 

Short chain fatty acids (SCFAs), such as acetic acid, butyric acid, and propionic acid, are physiologically active products of the commensal microbial fermentation of macronutrients in the colon [8]. Mounting evidence has demonstrated that SCFAs play important roles in pathological processes of systemic inflammation and diabetes by activating their receptors G-protein coupled receptors 41 and 43 [9,10]. Patients with diabetes exhibited lower contents of SCFAs [11] and the proportion of SCFAs-producing bacterial communities in the host gut, such as *Bifidobacterium*, *Bacteroidetes*, and *Lactobacillus* [12]. Butyrate supplementation ameliorated inflammation through modulating gut microbiota and preserving gut epithelial barrier function in db/db mice [13]. In addition, it has been widely reported that dietary intervention could modulate gut microbiota and SCFAs production, eventually attenuating low-grade inflammation and improving glucose metabolism [14,15].

Recently, a growing number of studies have proven that herbal medicines exerted beneficial effects against metabolic diseases for their abilities to modulate gut microbiota [15,16]. *Potentilla discolor* Bunge (PDB), a perennial herb with broad distribution in China, has been used as a traditional Chinese medicine in the therapy of many diseases, such as malaria, diarrhea, and ringworm [17]. It has been proven that the triterpenoid derivatives from PDB have remarkable anti-tumor activity, indicating that this herb is a potential anticancer agent [18]. In addition, clinical studies revealed that the whole herb of PDB ameliorated T2D via improving insulin resistance and protecting pancreatic β-cells from apoptosis [19]. Coincidentally, hypoglycemic activity of the extract from PDB was also confirmed in alloxan-induced diabetic mice [20]. Though many pharmacological studies have confirmed the therapeutic efficacy of PDB against T2D, it remains unclear whether this effect of PDB is associated with the modulation of the intestinal flora. Rodents with a combination of HFD and streptozotocin (STZ) treatment have been proposed as a desirable T2D model that imitates the natural history and metabolic features of T2D in humans, since HFD exposure is associated with obesity and insulin resistance and STZ injection induces pancreatic beta cells destruction [21]. On that basis, the present study aimed to explore the impact of PDB on the gut microbiota and inflammation status of diabetic mice induced by HFD and streptozotocin (STZ).

## 2. Materials and Methods

### 2.1. Materials and Regents

STZ was purchased from Sigma Chemicals (St. Louis, MO, USA). Primary antibodies for TLR4, NF-κB, ZO-1, Claudin3, Occludin, and β-actin were obtained from Bioss Biotechnology co. Ltd (Beijing, China). The normal chow diets containing 19.38% (w/w) protein, 45.79% (w/w) carbohydrate, and 4.48% (w/w) fat, and the high-fat diets containing 26.2% (w/w) protein, 26.30% (w/w) carbohydrate, and 34.90% (w/w) fat were purchased from KeAoXieLi Feed Co. Ltd (Beijing, China).

### 2.2. Preparation of Potentilla Discolor Bunge Water Extract

Air-dried *Potentilla discolor* Bunge was from Tang County, Hebei Province, China. The crushed whole herb of *Potentilla discolor* Bunge was extracted with ten volumes of deionized water for 1 h by heating to boiling under reflux. After cooling, the extract was filtered and the supernatant was collected. The reflux extraction process was repeated twice. The collections were combined and centrifuged (5000 g, 5 min). The supernatant was concentrated under reduced pressure at 40 °C and then lyophilized, and water extract of *Potentilla discolor* Bunge was obtained for further investigation. 

### 2.3. Animals and Experimental Design

**Figure 1:**
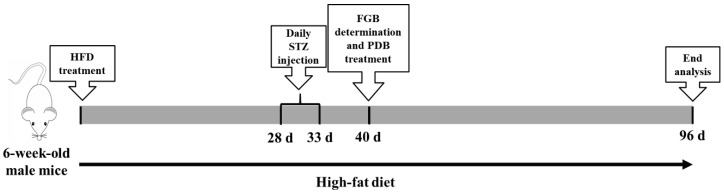
Outline of study design. After four weeks high-fat diet (HFD) administration, male C57BL/6J mice were daily injected with streptozotocin (STZ) at a dose of 40 mg/kg body weight for five days. At seven days following STZ treatment, the fasting blood glucose (FBG) was measured and the mice with FBG levels > 11.1 mmol/L were subjected daily to oral gavage of *Potentilla discolor* Bunge (PDB) (400 mg/kg body weight). After eight weeks administration, the effects of PDB on gut microbiota and inflammation of diabetic mice were evaluated.

Five-week-old male C57BL/6J mice (19 ± 1 g) were purchased from Vital River Laboratory Animal Center (Beijing, China). The animal experiments were carried out with approval of the Animal Experimentation Committee of China Agricultural University (Approval No. KY160018). Animals were housed at a temperature of 23 ± 1 °C in a 12 h daylight cycle and allowed indicated food and water *ad libitum*. The T2D model was established according to previous studies [22,23], and the outline of the study design is shown in Figure 1. Briefly, after seven days acclimatization, mice were randomly divided into two groups: the ND group (n = 6, fed with normal chow diets) and HFD group (n = 20, fed with HFD). After four weeks of respective diets, mice in the HFD group were continuously intraperitoneally injected with STZ dissolved in 0.1 mol/L citrate buffer (pH = 4.5) at 40 mg/kg body weight for five days, while the ND group were injected with vehicle at the same volume. At seven days following STZ treatment, the fasting blood glucose (FBG) was measured and the mice with FBG levels > 11.1 mmol/L were considered as diabetic mice [24]. The diabetic mice were given the same diet (HFD) and randomly divided into two groups of 6 animals each: T2D group and PDB group. Mice in the PDB group were subjected daily to oral gavage of PDB (400 mg/kg body weight), whereas mice in the control and T2D group were treated with the same volume of physiological saline. After treating for two months, fresh fecal samples were collected and frozen at −80 °C for microbiota analysis. Mice with 12 h fasting were euthanized and blood was collected. Serum was isolated for the quantification of inflammatory cytokines. The colon was carefully separated from the abdominal. After softly clearing out the stool with a phosphate buffered solution (PBS), the colon samples were then stored at −80 °C for western blot analysis. 

### 2.4. Plasma and Fecal LPS Measurement

The serum LPS and inflammatory cytokines, including tumor necrosis factor-α (TNF-α), interleukin-6 (IL-6), and interleukin-1β (IL-1β), were quantified by enzyme-linked immunosorbent assay (ELISA) kits (R&D, Minneapolis, MN, USA), according to the manufacturer’s instructions. 

The fecal LPS determination was performed as described in a previous report [25]. Briefly, fecal samples (100 mg) were homogenized in 50 mL PBS by sonication for 60 min on ice. Then the mixture was centrifugated at 4000 g for 10 min and the supernatant was collected. After sterilization with filtration, the concentration of LPS in the supernatant was measured using an ELISA kit (R&D, Minneapolis, MN, USA), according to the manufacturer’s protocol. 

### 2.5. Gut Microbiota Analysis by 16S rRNA High Throughput Sequencing

#### 2.5.1. Bacterial Genomic DNA Extraction

Genomic DNA in mice fecal samples was extracted using an E.Z.N.A.® Soil DNA kit (Omega Bio-tek, Inc., Norcross, GA, USA). All operations were followed according to the manufacturer’s instructions. 

#### 2.5.2. 16S rRNA Tagged Sequencing Amplification and Illumina MiSeq Sequencing

The hypervariable V3 to V4 regions of the 16S rRNA gene were amplified with barcoded primer 338F 5′-ACTCCTACGGGAGGCAGCAG-3′ and 806R 5′-GGACTACHVGGGTWTCTAAT-3′. The PCR reaction system and condition were performed in accordance with a previous study [24]. The PCR reaction was performed in a reaction volume of 25 μL, containing DNA template 10 ng, 5×FastPfu Buffer 4 μL, dNTPs 2 μL (2.5 mM), each paired primer 0.8 μL (5 μM) and Fast Pfu Polymerase 0.4 μL. The PCR reaction was carried out in a 96-well plate under the following procedure: 95 °C for 2 min; followed by 27 cycles 30 s at 95 °C, 30 s at 55 °C, 45 s at 72 °C; finally, 45 s at 72 °C. Then the resulting amplicons were extracted from 2% agarose gels and cleaned using AxyPrep DNA Gel Extraction kit (Axygen Biosciences, Union City, CA, USA). After quantification, PCR products were paired-end sequenced (2 × 300) using Illumina HiSeq platform in accordance with the manufacturer’s instructions.

#### 2.5.3. Bioinformatic Analysis

The lowest sequencing reads of all the samples (45,408 reads) were randomly selected for further analysis. Operational taxonomic units (OTUs) were obtained from 16S rRNA gene sequences using UPARSE (version 7.1 http://drive5.com/uparse/) with a threshold of 97% similarity. The taxonomic assignment of OTUs was carried out using RDP Classifier (http://rdp.cme.msu.edu/) against the silva (SSU115) 16S rRNA database. Alpha-diversity including Ace and Shannon index was analyzed with QIIME, while beta diversity was calculated with Principal Component Analysis (PCA) to further distinguish the between-sample diversity. Linear discriminant analysis effect size (LEfSe) was determined by a non-parametric factorial Kruskal-Wallis (KW) sum-rank test and linear discriminant analysis (LDA) was used to evaluate the effect size of corresponding differential species abundance. The correlations between gut microbiota and inflammation cytokines were carried out based on Spearman’s rho non-parametric correlation analysis.

### 2.6. Fecal SCFAs Analysis

Fecal SCFAs analysis was performed according to a previous report [26]. Briefly, feces were homogenized in deionized water and 5% (v/v) H_2_SO_4_ was added to acidify. After acidification, diethyl ether was added to extract the SCFAs and then centrifuged at 13200 g for 20 min to obtain the supernatant. SCFAs including acetic acid, propionic acid, and butyric acid were quantified using a gas chromatography-mass spectrometer (GCMS-QP2010 Ultra system, Shimadzu Corporation, Japan) equipped with a WAX capillary column (0.25 μm × 0.32 mm × 30 m). The carrier gas was helium at a rate of 1.0 mL/min. The injection and ionization temperatures were set as 180 and 230 °C, respectively. 

### 2.7. Quantitative Real-time PCR (qRT-PCR) Analysis

Total RNA of the colon tissue was isolated using Trizol regent (Tiangen Biotech, Beijing, China). cDNA was prepared using EasyScript Plus cDNA synthesis kit (ABM, Richmond, BC, Canada) following the manufacturer’s instructions. Synthesized cDNA was used as a template and quantified using a Techne Quantica real-time PCR detection system (Techne, Stone, Staffordshire, UK). The thermal cycling condition and reaction system for qRT-PCR analysis were based on a previous report [27]. Primer sequences for the target genes were: forward, 5′-CATCTTCTCAAAATTCGAGTGACAA-3′; reverse, 5′-TGGGAGTAGACAAGGTACAACCC-3′ for the TNF-α gene; forward, 5′- AACGATGATGCACTTGCAGA -3′; reverse, 5′- GAGCATTGGAAATTGGGGTA -3′ for the IL-6 gene; forward, 5′-AATCTCGCAGCAGCACATCAACA-3′; reverse, 5′- GTTGTTCATCTCGGAGCCTGTAGTG for the IL-1β gene; forward, 5′-TGGCAAAGTGGAGATTGTTGC-3′; reverse, 5′-AAGATGGTGATGGGCTTCCCG-3′ for the GADPH gene. The house-keeping gene GADPH was used as a reference.

### 2.8. Western Blot Analysis

Total protein was isolated from the colon tissue using lysis buffer (Beyotime, Biotech, Haimen, Jiangsu, China), then centrifuged (4 °C, 6300 g, 10 min) to collect the supernatant. Protein concentration of the supernatant was determined by BCA Protein Assay Reagent (Beyotime, Biotech, Haimen, Jiangsu, China). The 20 ug protein of each sample was separated by 10% SDS-PAGE, then transferred to 0.45 μm polyvinylidene fluoride (PVDF) membranes (Millipore, Billerica, MA, USA). After blocking with 5% (w/v) skimmed milk powder, the PVDF membranes were hybridized overnight with specific primary antibodies at 4 °C. After incubation with a secondary antibody, the PVDF membranes were added to an enhanced chemiluminescence (ECL) reagent to visualize the protein bands. The intensity of protein bands was quantified with Image J software (National Institutes of Health). β-actin levels were analyzed as internal controls.

### 2.9. Statistical Analysis

Data were present as the mean ± standard deviations (SD). Statistically significant differences among the different treatment groups were determined by one-way analysis of variance (ANOVA) followed by Tukey’s multiple comparison test using SPSS software (version 19.0, IBM Inc., Chicago, IL, USA). Correlations between parameters were analyzed using Spearman’s correlation test. *p* value < 0.05 were regarded statistically significant while *p* value < 0.01 were extremely significant. 

## 3. Results

### 3.1. Suppressive Effects of PDB on Systemic Endotoxin and Inflammation in HFD and STZ-treated Mice

**Figure 2:**
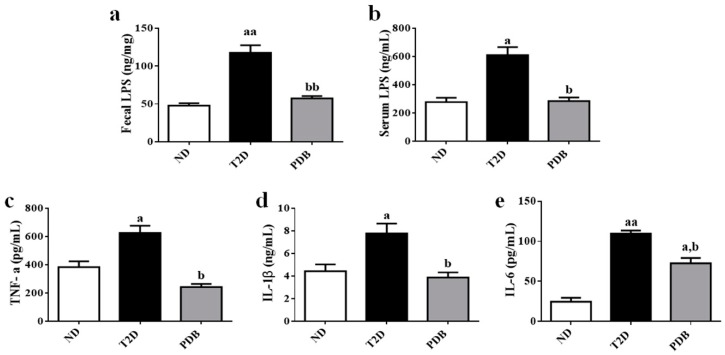
Effects of PDB on systemic endotoxemia and inflammation of type 2 diabetic (T2D) mice. The fecal lipopolysaccharide (LPS) (**a**), serum LPS (**b**), serum TNF-α (**c**), serum IL-1β (**d**), and IL-6 (**e**) were determined by ELISA kits. Data are presented as mean ± SD, n=6. ^a^ *p* < 0.05, ^aa^ *p* < 0.01 versus the normal diet (ND) group. ^b^ *p* < 0.05, ^bb^ *p* < 0.01 versus the T2D group.

In attempt to investigate the effects of PDB on endotoxemia and inflammation in diabetic mice, the levels of LPS and inflammatory cytokines including TNF-α, IL-1β, and IL-6 were measured. As shown in Figure 2a,b, there were increases in the contents of fecal and serum LPS in the T2D group as compared with the ND group (*p* < 0.01, *p* < 0.05). PDB intervention significantly decreased the contents of fecal and serum LPS in diabetic mice (*p* < 0.01, *p* < 0.05). In addition, levels of TNF-α, IL-1β, and IL-6 were significantly increased in diabetic mice (*p* < 0.05). However, PDB administration remarkably decreased the contents of TNF-α, IL-1β, and IL-6 in serum as compared with the T2D group (*p* < 0.05) (Figure 2c–e), indicating that PDB ameliorated systemic endotoxemia and inflammation in mice with T2D. 

### 3.2. Effects of PDB on Gut Microbiota Structure in Diabetic Mice

**Figure 3:**
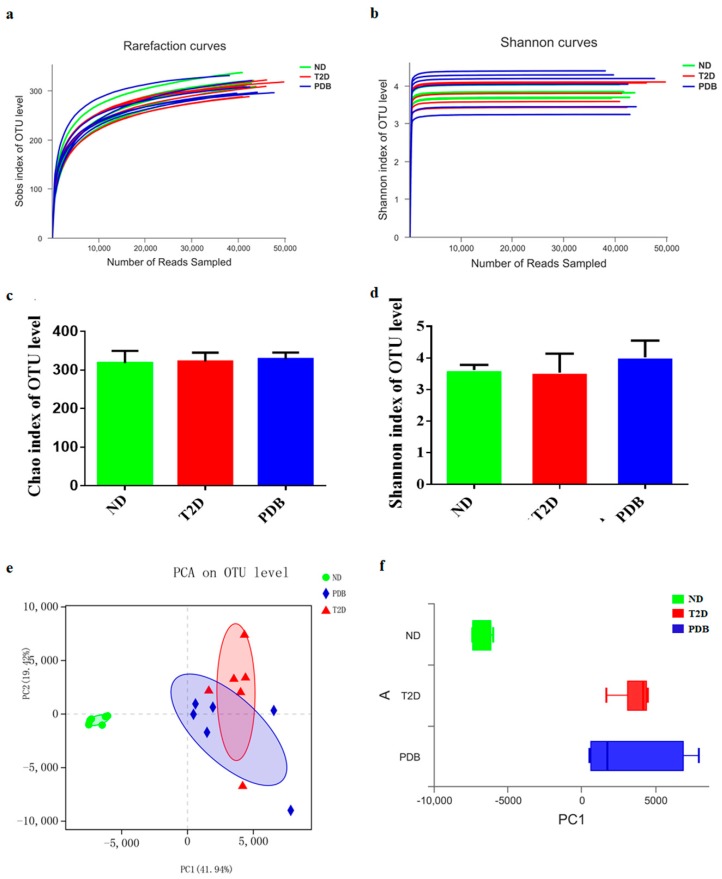
Effects of PDB on the diversity and structure of the gut microbiota in HFD and STZ-treated mice. Rarefaction curves of observed species from fecal samples including sobs index (**a**) and Shannon index (**b**). The α-diversity including Chao index (**c**) and Shannon index (**d**). The β-diversity presenting as principal component analysis PCA (**e**,**f**). There were six mice in each group.

To assess whether PDB administration is associated with modulation of gut microbiota structure in diabetic mice, the fecal samples were analyzed by 16S rRNA high throughput sequencing. A total of 778,121 effective reads were obtained from 18 fecal samples (*n* = 6 per group). The sobs and ShannonL141 rarefaction curves reached the plateau phase, thereby suggesting that the sequence depth captured was sufficient for the present study (Figure 3a,b). 

Chao1 and Shannon indices were determined to reflect the community richness and diversity, respectively. No obvious difference was observed in the Chao1 and Shannon index among these three groups (Figure 3c,d). Then PCA was carried out to further analyze the variation of gut flora structure. The mice in the T2D group formed a distinct bacterial community clustered separately from the normal mice. Meanwhile, the cluster formed by being administrated with PDB was similar to that of the T2D group (Figure 3e,f), indicating that PDB had little effect on overall structure alteration of gut microbiota in response to HFD and STZ treatment. 

### 3.3. Composition Changes of Gut Microbiota in Response to PDB Treatment

**Figure 4:**
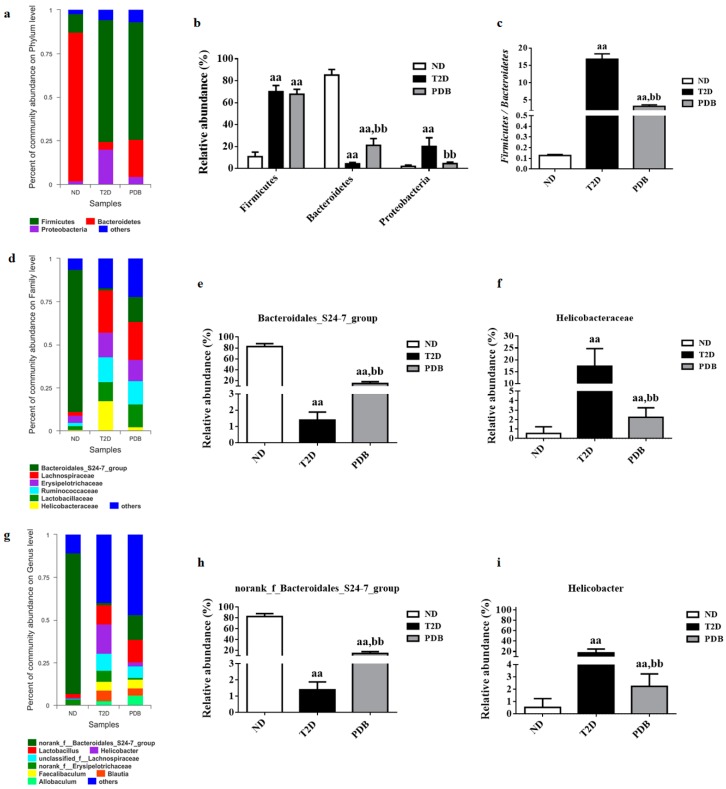
PDB changed gut microbiota composition of diabetic mice induced by HFD and STZ. Taxonomic distribution of bacterial communities of diabetic mice fecal samples at phylum level (**a**), family level (**d**) and genus level (**g**). The relative abundance of Firmicutes, Bacteroidetes, and Proteobacteria (**b**); The ratio of Firmicutes to Bacteroidetes (**c**); The relative abundance of Bacteroidales_S24-7_group (**e**), Helicobacteraceae (**f**), norank_f_Bacteroidales_S24-7_group (**h**), and Helicobacter (**i**). Data are presented as mean ± SD, n = 6. ^a^ *p* < 0.05, ^aa^ *p* < 0.01 versus the ND group. ^b^ *p* < 0.05, ^bb^ *p* < 0.01 versus the T2D group.

The relative abundance of bacterial phylotypes was compared between three groups to further investigate composition alterations in the gut microbiota. As presented in Figure 4a, the three most abundant bacterial phyla were determined in 18 fecal samples of three groups. Of these three bacteria, phyla including *Firmicutes* and *Proteobacteria* displayed higher abundances (*p* < 0.01), while the number of species of *Bacteroidetes* was lower after HFD and STZ treatment. PDB supplementation failed to modulate the abundance of *Firmicutes,* but significantly reversed the abundance change of *Bacteroidetes* in response to HFD and STZ treatment (Figure 4b, *p* < 0.01), resulting in a significantly lower ratio of *Firmicutes* and *Bacteroidetes* (Figure 4c, *p* < 0.01). In addition, PDB administration reduced the relative abundance of *Proteobacteria* in diabetic mice (Figure 4b, *p* < 0.01). At a family level, gut bacteria from the T2D group displayed an increased relative abundance of *Helicobacteraceae*, *Lachnospiraceae*, and *Ruminococcaceae* as well as a decreased relative abundance of *Bacteroidales_S24-7_group* when compared with the ND group (Figure 4d, *p* < 0.01). However, PDB administration had a notable enriching effect on the *Bacteroidales_S24-7_group* (Figure 4e, *p*<0.01), but a suppressing effect on *Helicobacteraceae* (Figure 4f, *p* < 0.01). At genus level, mice in the T2D group had a higher relative abundance of *unclassified_f_Lachnospiraceae*, *Ruminiclostridium*, *Allobaculum*, *Romboutsia*, *Anaerotruncus*, and *Helicobacter* (Figure 4g, *p* < 0.01), but a lower relative abundance of *norank_f_Bacteroidales_S24-7_group* compared to the ND group. Conversely, the lowered abundance of *norank_f_Bacteroidales_S24-7_group* was significantly elevated by PDB treatment (Figure 4h, *p* < 0.01). Meanwhile, the abundance of *Helicobacter* in diabetic mice was also significantly decreased after two months treatment of PDB (Figure 4i, *p* < 0.01). 

### 3.4. Modulation of Taxonomic Diversity by PDB in HFD and STZ-treated Mice

**Figure 5:**
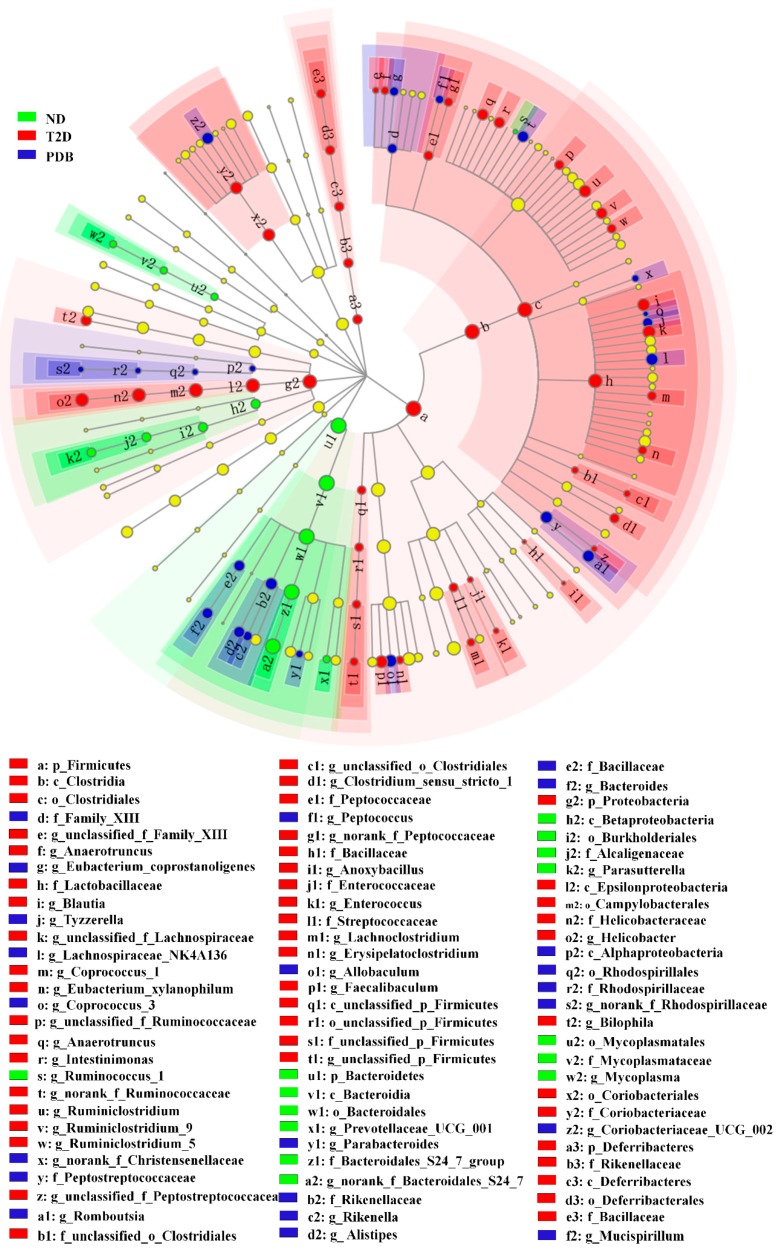
PDB intervention modulated taxonomic diversity of gut microbiota in diabetic mice. The cladogram showed gut bacterial taxa with a linear discriminant analysis (LDA) score > 3.0. There were six mice in each group.

The bacterial composition of three groups was further compared using LEfSe software to determine specific species in every treatment. As shown in Figure 5, the constitution of intestinal bacteria from fecal samples significantly varied in response to different diet treatment. Six significant different bacterial genera in ND group mainly belonged to *Bacteroidetes* and *Proteobacteria*. HFD and STZ treatments induced different changes in phylum *Firmicutes*, *Proteobacteria*, and *Deferribacteres*, containing 21 key genera. Fifteen major genera of *Firmicutes*, *Bacteroidetes*, and *Proteobacteria* with LDA score > 3.0 were restored by PDB administration, including *Parabacteroides*, *Eubacterium_nodatum_group*, *norank_f_Rhodospirillaceae*, *Tyzzerella*, *Rikenella*, *Alistipes*, *Lachnospiraceae_NK4A136_group*, *norank_f_Ruminococcaceae*, *Romboutsia*, *Coriobacteriaceae_UCG_002*, *Bacteroides*, *Allobaculum*, *Coprococcus_3*, and *norank_f_Christensenellaceae*.

### 3.5. Key Gut Microbiota Associated with FBG and Inflammatory Cytokines in Diabetic Mice

**Figure 6:**
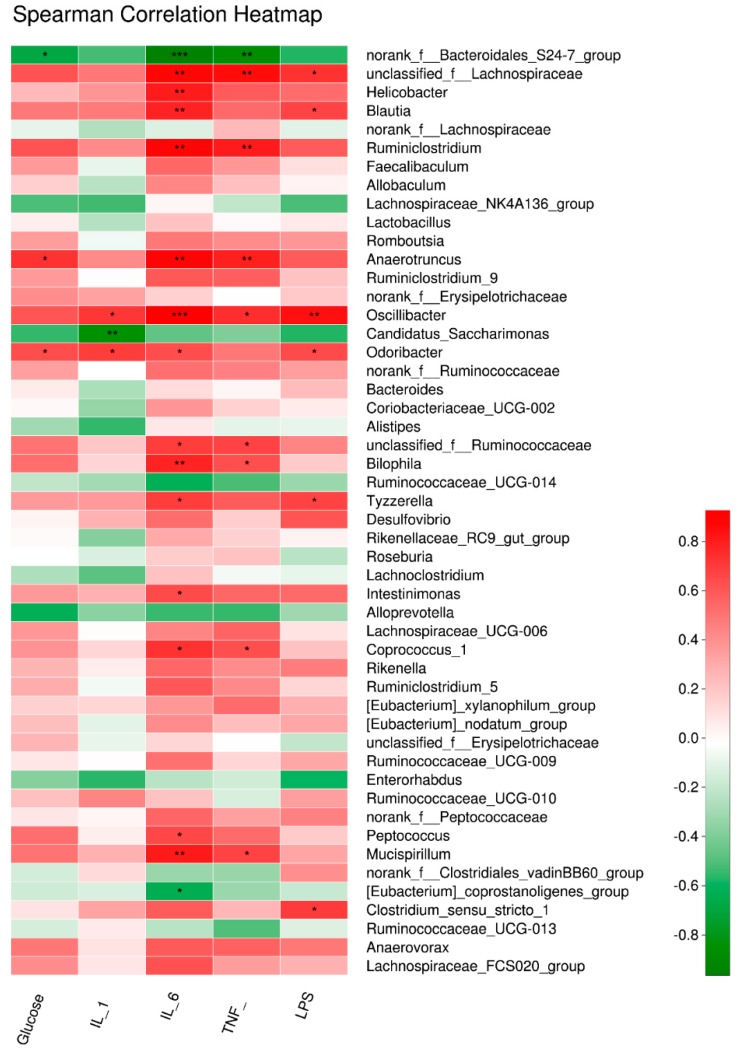
Correlation analysis between T2D-related biochemical parameters and the relative abundance of gut microbiota at genus level. A positive correlation was represented by red while a negative correlation was shown in green in the heap map. Statistical significance was calculated by Spearman’s rho non-parametric correlation analysis (* *p* < 0.05, ** *p* < 0.01, *** *p* < 0.001).

To further assess the potential relationship between variation of gut microbiota and inflammation induced by T2D, the correlation between 50 major genera obtained in the current study and inflammation-related parameters were calculated based on the Spearman’s rho non-parametric correlation analysis. As shown in Figure 6, the relative abundance of *unclassified_f_Lachnospiraceae* and *oscillibacter* was positively related to serum LPS and proinflammatory cytokines (*p* < 0.01). Furthermore, *Odoribacter* and *Anaerotruncus* were positively correlated with FBG and inflammation (*p* < 0.05). On the contrary, the genus of *norank_f__Bacteroidales_S24-7_group* exhibited a negative relationship with the inflammatory status and FBG (*p* < 0.05, *p* < 0.01), and *Candidatus_Saccharimonas* was negatively related to the content of IL-1β (*p* < 0.01). 

### 3.6. PDB Improved Intestinal Inflammation and Mucosal Barrier Function in HFD and STZ-Treated Mice

**Figure 7:**
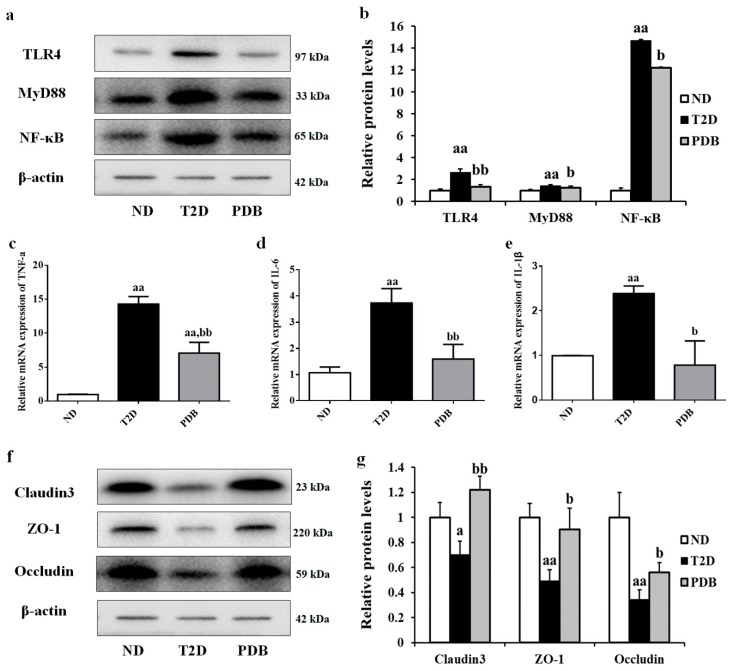
Effects of PDB on intestinal inflammation and intestinal mucosal barrier function of diabetic mice. The protein expression of TLR4, MyD88, NF-κB (**a**,**b**), Claudin3, ZO-1, and Occludin (**f**,**g**) in colon tissue was determined by western blot. TNF-α (**c**), IL-6 (**d**), and IL-1β (**e**) mRNA expression was measured by qRT-PCR. Data are presented as mean ± SD, *n* = 6. ^a^ *p* < 0.05, ^aa^ *p* < 0.01 versus the ND group. ^b^ *p* < 0.05, ^bb^ *p* < 0.01 versus the T2D group.

To investigate the effect of PDB on intestinal inflammation of diabetic mice, the expression of TLR4 and downstream MyD88 and NF-κB was detected (Figure 7a). HFD and STZ treatment caused a notable increase in TLR4, MyD88, and NF-κB expression in the colon tissue. As we expected, PDB administration significantly decreased transcription level of TLR4, MyD88, and NF- κB. In addition, elevated mRNA expression of proinflammatory cytokines TNF-α, IL-6, and IL-1β in the colon tissue of diabetic mice, was also significantly reversed by PDB administration (Figure 7b–d). All these results indicated that PDB notably attenuated intestinal inflammation in diabetic mice. 

Next, the effect of PDB on the protein expression of major tight junction proteins including Claudin3, ZO-1, and Occludin was evaluated. As shown in Figure 7e and f, a notable decrease in protein expression of Claudin3, ZO-1, and Occludin in the colon tissue of diabetic mice was observed (*p* < 0.05, vs. the ND group). However, PDB significantly improved gut integrity of diabetic mice as indicated by the elevated protein expression of Claudin3, ZO-1, and Occludin (*p* < 0.05). 

### 3.7. Effects of PDB Supplementation on the SCFAs Levels and Protein Expression of GPR41 and GPR43

**Figure 8:**
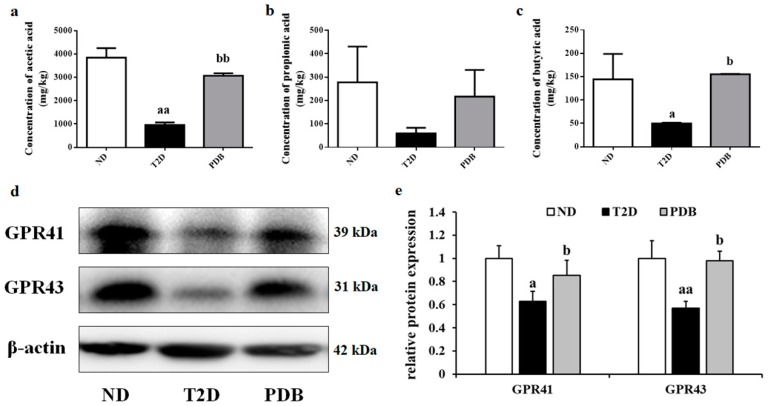
Effects of PDB supplementation on the SCFAs levels and protein expression of GPR41 and GPR43 in diabetic mice. Acetic acid (**a**), propionic acid (**b**), and butyric acid (**c**) content of feces samples were detected by GC-MS. The protein expression of GPR41 and GPR43 in colon tissue was determined by western blot (**d**,**e**). Data are presented as mean ± SD, ^a^ *p* < 0.05, ^aa^ *p* < 0.01 versus the ND group. ^b^ *p* < 0.05, ^bb^ *p* < 0.01 versus the T2D group.

HFD and STZ treatment led to a notable decrease in acetic acid and butyric acid content in diabetic mice feces, while mice with PDB administration showed significant recovery of acetic acid and butyric acid as compared with the T2D group (Figure 8a,c). However, there was no significant difference in the propionic acid level among ND, T2D, and PDB groups (Figure 8b). To assess the effects of PDB on SCFAs receptors, the protein expression of GPR41 and GPR43 was detected. As shown in Figure 8d,e, mice in the T2D group showed a significant decrease in the protein expression of GPR41 and GPR43 as compared with the ND group (*p* < 0.05). However, administration of PDB notably reversed this adverse trend to some extent (*p* < 0.05), indicating that PDB exerted beneficial effects on diabetic mice partly through regulating SCFAs levels and its receptors expression. 

## 4. Discussion

T2D is known to be a symptom of chronic low-grade inflammation. Pro-inflammatory cytokines elevated in individuals with T2D contribute to pancreatic beta cells apoptosis and insulin resistance, thereby exerting an important role in the onset and development of T2D [28]. LPS is regarded as a crucial trigger in pro-inflammation responses with increased synthesis and secretion of inflammatory cytokines through activating TLR4 and downstream transcription factor, including MyD88 and NF-κB [29]. Inflammation and LPS-induced insulin resistance were improved when TLR4 was inhibited [30]. Our study demonstrated that PDB significantly improved endotoxemia and inflammation of diabetic mice, evidenced by reduced contents of LPS and pro-inflammatory cytokines (including TNF-α, IL-6, and IL-1β) in serum, suppression in mRNA expression of TNF-α, IL-6, and IL-1β, as well as protein expression of TLR4, MyD88, and NF-κB in colon.

The crucial roles of gut microbiota in the etiology and development of various physiological diseases, such as inflammatory bowel disease, obesity, and T2D, have been widely recognized [31]. Gut microbiota is proposed as a potential diagnostic and therapeutic target for chronic metabolic diseases [32]. Increasing evidence demonstrated that herbal medicines modulated gut microbiota, thereby ameliorating inflammatory status and metabolic syndrome [33]. In the present study, PDB intervention did not change α-diversity and β-diversity of fecal bacteria in HFD and STZ treated mice, indicating that the overall structure of diabetic mice gut flora remained stable during the intervention of PDB. However, the ratio of *Firmicutes* and *Bacteroidetes* and relative abundance of *Proteobacteria* were significantly altered by PDB. This result is similar to the previous reports that capsaicin changed gut microbiota composition at phylum level, but no alteration in α-diversity and β-diversity [34,35]. 

*Firmicutes* and *Bacteroidetes* are considered as the predominant phyla in the host microbiota [36], and the ratio of *Firmicutes*/*Bacteroidetes* in the fecal bacteria is considered as a gauge of the overall gut microbiota balance [34]. It has been identified that the ratio of *Firmicutes* to *Bacteroidetes* is positively correlated to FBG and inflammation status [37]. Compared with the normal mice, T2D mice induced by HFD and STZ exhibited high contents of pro-inflammatory cytokines and insulin resistance, along with a larger proportion of *Firmicutes* and relatively less *Bacteroidetes* in the gut bacteria [38]. Song et al., reported that ingestion of red pitaya betacyanins prevented the elevation of FBG and serum inflammatory factors with the decreased ratio of *Firmicutes* to *Bacteroidetes* in diabetic mice [10]. Consistent with these reports, PDB showed no significant effect on the relative abundance of *Firmicutes*, but a reverting influence on the *Bacteroidetes* population (partly due to an increase in *norank_f_Bacteroidales_S24-7_group*), contributing to a lower ratio of *Firmicutes* to *Bacteroidetes*. *Proteobacteria*, less abundant than *Firmicutes* and *Bacteroidetes*, is significantly enriched in the gut flora of individuals with obesity and T2D [39]. *Proteobacteria* is proposed to be a potential pro-inflammatory phylum and closely correlated to the glucose homeostasis in T2D [40,41]. In the current study, PDB administration notably decreased the relative abundance of *Proteobacteria*, which was partly ascribed to a significant reduction in *Helicobacter*. The association between *Helicobacter* and T2D has been proposed, since *Helicobacter* affected oxidative stress and the absorption of glucose in diabetic individuals [42]. Based on these findings, we believed that the suppressive effect of PDB on inflammation in diabetic mice was associated with the alteration of gut microbiota composition, which was further proved by the results of Spearman’s rho non-parametric correlation analysis. 

The tight junction of intestinal mucosal cells is the primary barrier to prevent excessive leakiness of endotoxins and other noxious agents into the circulation system, which attenuates the activation of the immune system and inflammatory responses [43]. Disruption of the gut barrier is closely associated with the pathogenesis of various diseases such as T2D [44]. Gut microbes play important roles in regulating gut barrier function via modulation of epithelial tight junction proteins, such as Claudin3, ZO-1, and Occludin [5]. Diabetic mice induced by HFD and STZ showed dysbiosis of gut microbiota, down-regulated protein expression of Claudins, ZO-1, and Occludin, as well as impaired intestinal barrier function [45]. Thus, we speculated that the elevated protein expression of Claudin3, ZO-1, and Occludin by PDB treatment in diabetic mice partly contributed to the lower contents of LPS and pro-inflammatory cytokines in serum. 

It is well known that SCFAs exert a protective effect on inflammation and insulin resistance partly through GPR 41 and GPR43 mediated signaling pathways [46]. Diabetic rodents induced by HFD and STZ exhibited a lower relative abundance of SCFAs-producing bacteria, such as *Bacteroidales S24-7*, *Bacteroides,* resulting in decreased acetic acid and total SCFAs levels [47]. Oral butyrate administration significantly alleviated diabetic-endotoxemia with improved gut integrity and decreased ratio of *Firmicutes/Bacteroidetes* [13]. Mice lacking GPR41 and GPR43 showed a reduction in SCFAs-induced glucagon-like peptide (GLP-1) release, impaired glucose tolerance [48] as well as increased inflammation in white adipose [49]. *Lactobacillus* intervention not only decreased LPS and inflammatory cytokines secretion, but also parallelly enriched SCFAs-producing bacteria as well as activated GPR43. Besides, GPR41 and GPR43 also contributed to the protection of normal intestinal mucosal barrier function [32]. Inhibitors of GPRs attenuated the protective effect of acetate on colonic epithelial permeability [44]. In our study, PDB significantly elevated acetic acid and butyric acid levels of diabetic mice feces. The SCFAs receptors of colon tissue, GPR41, and GPR43, were also significantly activated by PDB administration. Additionally, *Bacteroides*, a SCFAs-producing bacterium, was enriched by PDB treatment. All these results suggested that the anti-inflammatory effects of PDB were partly ascribed to the increased production of SCFAs as well as the activation of related receptors GPR41 and GPR43.

In summary, PDB ameliorated endoxemia and inflammation in HFD and STZ-induced diabetic mice. The underlying mechanism was associated with the modulation of the gut microbiota composition, which subsequently contributed to improvement of inflammation and mucosal barrier function of colon, increased production of SCFAs, and activation of GPR41, and GPR43. However, the regulatory effects of PDB on gut microbiota are only proven in HFD and STZ induced diabetic model, which may need to be further investigated in other diabetic models. Additionally, the exact bioactive components of PDB and the underlying mechanism in regulating gut microbiota balance are supposed to be further clarified. Nonetheless, the current study provides crucial evidence to indicate that PDB might serve as a potential functional ingredient against diabetes and related inflammation. 

## References

[1] Zhang L., Qin Q., Liu M., Zhang X., He F., Wang G. (2018). Akkermansia muciniphila can reduce the damage of gluco/lipotoxicity, oxidative stress and inflammation, and normalize intestine microbiota in streptozotocin-induced diabetic rats. Pathog. Dis..

[2] Crommen S., Simon M.C. (2017). Microbial Regulation of Glucose Metabolism and Insulin Resistance. Genes.

[3] Larsen N., Vogensen F.K., van den Berg F.W.J., Nielsen D.S., Andreasen A.S., Pedersen B.K., Abu Al-Soud W., Sorensen S.J., Hansen L.H., Jakobsen M. (2010). Gut Microbiota in Human Adults with Type 2 Diabetes Differs from Non-Diabetic Adults. PLoS ONE.

[4] Sircana A., Framarin L., Leone N., Berrutti M., Castellino F., Parente R., De Michieli F., Paschetta E., Musso G. (2018). Altered Gut Microbiota in Type 2 Diabetes: Just a Coincidence?. Curr. Diabetes Rep..

[5] Tran C.D., Grice D.M., Wade B., Kerr C.A., Bauer D.C., Li D.M., Hannan G.N. (2015). Gut permeability, its interaction with gut microflora and effects on metabolic health are mediated by the lymphatics system, liver and bile acid. Future Microbiol..

[6] Daniel H., Gholami A.M., Berry D., Desmarchelier C., Hahne H., Loh G., Mondot S., Lepage P., Rothballer M., Walker A. (2014). High-fat diet alters gut microbiota physiology in mice. SME J..

[7] Shin N.R., Lee J.C., Lee H.Y., Kim M.S., Whon T.W., Lee M.S., Bae J.W. (2014). An increase in the *Akkermansia* spp. population induced by metformin treatment improves glucose homeostasis in diet-induced obese mice. Gut.

[8] McLoughlin R.F., Berthon B.S., Jensen M.E., Baines K.J., Wood L.G. (2017). Short-chain fatty acids, prebiotics, synbiotics, and systemic inflammation: A systematic review and meta-analysis. Am. J. Clin. Nutr..

[9] Trompette A., Gollwitzer E.S., Yadava K., Sichelstiel A.K., Sprenger N., Ngom-Bru C., Blanchard C., Junt T., Nicod L.P., Harris N.L. (2014). Gut microbiota metabolism of dietary fiber influences allergic airway disease and hematopoiesis. Nat. Med..

[10] Song H., Chu Q., Yan F., Yang Y., Han W., Zheng X. (2016). Red pitaya betacyanins protects from diet-induced obesity, liver steatosis and insulin resistance in association with modulation of gut microbiota in mice. J. Gastroenterol. Hepatol..

[11] Zhu K.X., Nie S.P., Tan L.H., Li C., Gong D.M., Xie M.Y. (2016). A Polysaccharide from Ganoderma atrum Improves Liver Function in Type 2 Diabetic Rats via Antioxidant Action and Short-Chain Fatty Acids Excretion. J. Agric. Food Chem..

[12] Conlon M.A., Bird A.R. (2015). The Impact of Diet and Lifestyle on Gut Microbiota and Human Health. Nutrients.

[13] Xu Y.H., Gao C.L., Guo H.L., Zhang W.Q., Huang W., Tang S.S., Gan W.J., Xu Y., Zhou H., Zhu Q. (2018). Sodium butyrate supplementation ameliorates diabetic inflammation in db/db mice. J. Endocrinol..

[14] Houghton D., Hardy T., Stewart C., Errington L., Day C.P., Trenell M.I., Avery L. (2018). Systematic review assessing the effectiveness of dietary intervention on gut microbiota in adults with type 2 diabetes. Diabetologia.

[15] Tong X., Xu J., Lian F., Yu X., Zhao Y., Xu L., Zhang M., Zhao X., Shen J., Wu S. (2018). Structural Alteration of Gut Microbiota during the Amelioration of Human Type 2 Diabetes with Hyperlipidemia by Metformin and a Traditional Chinese Herbal Formula: A Multicenter, Randomized, Open Label Clinical Trial. mBio.

[16] Wang J.H., Bose S., Lim S.K., Ansari A., Chin Y.W., Choi H.S., Kim H. (2017). Houttuynia cordata Facilitates Metformin on Ameliorating Insulin Resistance Associated with Gut Microbiota Alteration in OLETF Rats. Genes.

[17] Zhang L., Yang J., Chen X.Q., Zan K., Wen X.D., Chen H., Wang Q., Lai M.X. (2010). Antidiabetic and antioxidant effects of extracts from Potentilla discolor Bunge on diabetic rats induced by high fat diet and streptozotocin. J. Ethnopharmacol..

[18] Zhang J., Liu C., Huang R.Z., Chen H.F., Liao Z.X., Sun J.Y., Xia X.K., Wang F.X. (2017). Three new C-27-carboxylated-lupane-triterpenoid derivatives from Potentilla discolor Bunge and their in vitro antitumor activities. PLoS ONE.

[19] Tomczyk M., Latte K.P. (2009). Potentilla—A review of its phytochemical and pharmacological profile. J. Ethnopharmacol..

[20] Yang J., Chen H., Zhang L., Wang Q., Lai M.-X. (2009). Anti-diabetic effect of standardized extract ofPotentilla discolorBunge and identification of its active components. Drug Dev. Res..

[21] Srinivasan K., Viswanad B., Asrat L., Kaul C.L., Ramarao P. (2005). Combination of high-fat diet-fed and low-dose streptozotocin-treated rat: A model for type 2 diabetes and pharmacological screening. Pharmacol. Res..

[22] Gilbert E.R., Fu Z., Liu D. (2011). Development of a nongenetic mouse model of type 2 diabetes. Exp. Diabetes Res..

[23] Li X., Sui Y., Li S., Xie B., Sun Z. (2016). A-type procyanidins from litchi pericarp ameliorate hyperglycaemia by regulating hepatic and muscle glucose metabolism in streptozotocin (STZ)-induced diabetic mice fed with high fat diet. J. Funct. Foods.

[24] You Y., Ren T., Zhang S., Shirima G.G., Cheng Y., Liu X. (2015). Hypoglycemic effects of Zanthoxylum alkylamides by enhancing glucose metabolism and ameliorating pancreatic dysfunction in streptozotocin-induced diabetic rats. Food Funct..

[25] Li T., Gao J., Du M., Mao X. (2018). Milk fat globule membrane supplementation modulates the gut microbiota and attenuates metabolic endotoxemia in high-fat diet-fed mice. J. Funct. Foods.

[26] Wang G., Li X., Zhao J., Zhang H., Chen W. (2017). Lactobacillus casei CCFM419 attenuates type 2 diabetes via a gut microbiota dependent mechanism. Food Funct..

[27] Gao J., Song J., Du M., Mao X. (2018). Bovine alpha-Lactalbumin Hydrolysates (alpha-LAH) Ameliorate Adipose Insulin Resistance and Inflammation in High-Fat Diet-Fed C57BL/6J Mice. Nutrients.

[28] Gomes J.M.G., Costa J.A., Alfenas R.C.G. (2017). Metabolic endotoxemia and diabetes mellitus: A systematic review. Metabolism: Clin. Exp..

[29] Kim S.H., Bang J., Son C.N., Baek W.K., Kim J.M. (2018). Grape seed proanthocyanidin extract ameliorates murine autoimmune arthritis through regulation of TLR4/MyD88/NF-kappaB signaling pathway. Korean J. Intern. Med..

[30] Liang H.Y., Hussey S.E., Sanchez-Avila A., Tantiwong P., Musi N. (2013). Effect of Lipopolysaccharide on Inflammation and Insulin Action in Human Muscle. PLoS ONE.

[31] Pascale A., Marchesi N., Marelli C., Coppola A., Luzi L., Govoni S., Giustina A., Gazzaruso C. (2018). Microbiota and metabolic diseases. Endocrine.

[32] Zhang Y., Tang K., Deng Y., Chen R., Liang S., Xie H., He Y., Chen Y., Yang Q. (2018). Effects of shenling baizhu powder herbal formula on intestinal microbiota in high-fat diet-induced NAFLD rats. Biomed. Pharmacother..

[33] Wang J.H., Bose S., Kim G.C., Hong S.U., Kim J.H., Kim J.E., Kim H. (2014). Flos Lonicera Ameliorates Obesity and Associated Endotoxemia in Rats through Modulation of Gut Permeability and Intestinal Microbiota. PLoS ONE.

[34] Song J.X., Ren H., Gao Y.F., Lee C.Y., Li S.F., Zhang F., Li L., Chen H. (2017). Dietary Capsaicin Improves Glucose Homeostasis and Alters the Gut Microbiota in Obese Diabetic ob/ob Mice. Front. Physiol..

[35] Kang C., Zhang Y., Zhu X.H., Liu K., Wang X.L., Chen M.T., Wang J., Chen H., Hui S.C., Huang L. (2016). Healthy Subjects Differentially Respond to Dietary Capsaicin Correlating with Specific Gut Enterotypes. J. Clin. Endocrinol. Metab..

[36] Li X., Watanabe K., Kimura I. (2017). Gut Microbiota Dysbiosis Drives and Implies Novel Therapeutic Strategies for Diabetes Mellitus and Related Metabolic Diseases. Front. Immunol..

[37] Yan H., Lu J., Wang Y., Gu W., Yang X., Yu J. (2017). Intake of total saponins and polysaccharides from Polygonatum kingianum affects the gut microbiota in diabetic rats. Phytomedicine.

[38] Zhu Y., Bai J., Zhang Y., Xiao X., Dong Y. (2016). Effects of bitter melon (*Momordica charantia* L.) on the gut microbiota in high fat diet and low dose streptozocin-induced rats. Int. J. Food Sci. Nutr..

[39] Zhang Q., Yu H., Xiao X., Hu L., Xin F., Yu X. (2018). Inulin-type fructan improves diabetic phenotype and gut microbiota profiles in rats. PeerJ.

[40] Choi Y., Kwon Y., Kim D.K., Jeon J., Jang S.C., Wang T., Ban M., Kim M.H., Jeon S.G., Kim M.S. (2015). Gut microbe-derived extracellular vesicles induce insulin resistance, thereby impairing glucose metabolism in skeletal muscle. Sci. Rep..

[41] Graessler J., Qin Y., Zhong H., Zhang J., Licinio J., Wong M.L., Xu A., Chavakis T., Bornstein A.B., Ehrhart-Bornstein M. (2013). Metagenomic sequencing of the human gut microbiome before and after bariatric surgery in obese patients with type 2 diabetes: Correlation with inflammatory and metabolic parameters. Pharmacogenomics J..

[42] Xie Y., Xiao M., Ni Y., Jiang S., Feng G., Sang S., Du G. (2018). Alpinia oxyphylla Miq. Extract Prevents Diabetes in Mice by Modulating Gut Microbiota. J. Diabetes Res..

[43] Balakumar M., Prabhu D., Sathishkumar C., Prabu P., Rokana N., Kumar R., Raghavan S., Soundarajan A., Grover S., Batish V.K. (2018). Improvement in glucose tolerance and insulin sensitivity by probiotic strains of Indian gut origin in high-fat diet-fed C57BL/6J mice. Eur. J. Nutr..

[44] Suzuki T., Yoshida S., Hara H. (2008). Physiological concentrations of short-chain fatty acids immediately suppress colonic epithelial permeability. Br. J. Nutr..

[45] Gu J.F., Su S.L., Guo J.M., Zhu Y., Zhao M., Duan J.A. (2017). The aerial parts of Salvia miltiorrhiza Bge. strengthen intestinal barrier and modulate gut microbiota imbalance in streptozocin-induced diabetic mice. J. Finct. Foods.

[46] Saad M.J., Santos A., Prada P.O. (2016). Linking Gut Microbiota and Inflammation to Obesity and Insulin Resistance. Physiology.

[47] Zhang B., Sun W., Yu N., Sun J., Yu X., Li X., Xing Y., Yan D., Ding Q., Xiu Z. (2018). Anti-diabetic effect of baicalein is associated with the modulation of gut microbiota in streptozotocin and high-fat-diet induced diabetic rats. J. Funct. Foods.

[48] Tolhurst G., Heffron H., Lam Y.S., Parker H.E., Habib A.M., Diakogiannaki E., Cameron J., Grosse J., Reimann F., Gribble F.M. (2012). Short-Chain Fatty Acids Stimulate Glucagon-Like Peptide-1 Secretion via the G-Protein-Coupled Receptor FFAR2. Diabetes.

[49] Kim S., Kim J.H., Park B.O., Kwak Y.S. (2014). Perspectives on the therapeutic potential of short-chain fatty acid receptors. BMB Rep..

